# The Effects of Running Compared with Functional High-Intensity Interval Training on Body Composition and Aerobic Fitness in Female University Students

**DOI:** 10.3390/ijerph182111312

**Published:** 2021-10-28

**Authors:** Yining Lu, Huw D. Wiltshire, Julien S. Baker, Qiaojun Wang

**Affiliations:** 1 Cardiff School of Sport and Health Sciences, Cardiff Metropolitan University, Cardiff CF5 2YB, UK; st20184530@outlook.cardiffmet.ac.uk (Y.L.); hwiltshire@cardiffmet.ac.uk (H.D.W.); 2 Department of Sport, Physical Education and Health, Hong Kong Baptist University, Kowloon Tong, Hong Kong; jsbaker@hkbu.edu.hk; 3 Faculty of Sport Science, Ningbo University, Ningbo 315000, China

**Keywords:** high-intensity interval training, high-intensity functional training, body composition, aerobic fitness, muscle performance

## Abstract

High-intensity interval running (HIIT-R) and high-intensity functional training (HIFT) are two forms of HIIT exercise that are commonly used. The purpose of this study was to determine the effects of HIFT on aerobic capacity and body composition when compared to HIIT-R in females. Twenty healthy, untrained female university students (age 20.5 ± 0.7 year) were randomly assigned to a 12-week HIIT-R or HIFT intervention. The HIIT-R group involved a 30 s maximal shuttle run with a 30 s recovery period, whereas the HIFT involved multiple functional exercises with a 2:1 work-active recovery ratio. Body composition, VO_2_max, and muscle performance were measured before and post intervention. As a result, HIIT-R and HIIT-F stimulated similar improvements in VO_2_max (17.1% ± 5.6% and 12.7% ± 6.7%, respectively, *p* > 0.05). Only the HIIT-F group revealed significant improvements in muscle performance (sit-ups, 16.5% ± 3.1%, standing broad jump 5.1% ± 2.2%, *p* < 0.05). Body fat percentage decreased (17.1% ± 7.4% and 12.6% ± 5.1%, respectively, *p* < 0.05) in both HIIT-R and HIIT-F with no between-group differences. We concluded that HIFT was equally effective in promoting body composition and aerobic fitness compared to HIIT-R. HIFT resulted in improved muscle performance, whereas the HIIT-R protocol demonstrated no gains.

## 1. Introduction

Regular physical activity (PA) is beneficial for health [1,2,3]. Despite the well documented benefits of moderate- to vigorous-intensity PA, 31% of adults worldwide do not engage in sufficient PA for health benefits as recommended by the World Health Organization (WHO) and the American College of Sports Medicine (ACSM) [4,5,6]. Frequently reported barriers to physical activity are physical exertion, time, and financial expenditure [7,8]. Thus, compared to traditional continuous training, which is characterized by long-duration, continuous aerobic exercises, and moderate-intensities, high-intensity interval training (HIIT) appears to be an efficient pathway to enhance PA and improve health [9].

HIIT involves repeated bouts of high-intensity exercises separated by a recovery using low-intensity activities or inactivity [10]. Recent studies had indicated that HIIT has a similar, or even greater positive, effect on physical fitness, especially on body composition and cardiorespiratory health [11,12,13,14]. From a time/benefit perspective, HIIT appears to help physically inactive individuals overcome a major time and participation barrier to maintaining a healthier lifestyle [15].

Originally, HIIT was used to improve the performance of endurance athletes [16]. Cycling, running, and rowing are traditional exercise modalities that adopted the use of HIIT protocols, while for individuals who perform exercise for health and recreation, these traditional modalities seem boring and do not engage individuals because of the repetitive nature of the exercise combined with repetition. This is considered as a negative impact for maintaining regular exercise and has been cited as “lack of enjoyment” when investigating barriers to exercise [17].

The intrinsic factors of participants are also important when considering exercise adherence [18,19]. Several studies have revealed that adherence is affected by exercise intensity, especially among inactive individuals [20,21].

High-intensity functional training (HIFT) has become a relatively popular training modality in recent years and is an alternative to traditional aerobic activities. The HIFT protocol consists of a variety of functional movements that are executed at a high intensity [22,23]). Recently, several investigators have studied the effects of HIFT on physical fitness promotion. After engaging in HIFT protocols, participants show significant improvements in cardiorespiratory fitness [24,25] and body composition [25,26]. Providing similar or greater health promotions compared to moderate-intensity continuous training, HIFT demonstrates further improvements in muscle fitness [27,28]. Additionally, participants perceive this type of activity to be more enjoyable when engaging in HIFT compared to those individuals performing traditional HIIT [29,30]. Moreover, most HIFT protocols are executed using the participant’s own body weight, allowing the participant to control the exercise intensity. This helps to improve exercise adherence [7,19,20].

Although studies have shown that HIFT has similar or superior benefits for physical fitness compared to moderate-intensity continuous training and have indicated more enjoyment compared to HIIT, the question remains as to whether HIFT is as efficient as HIIT for improving health-related fitness.

While HIFT is not synonymous with HIIT, they share an important conceptual commonality in the modality of both being of a high intensity. The current study was undertaken to clarify how a functional exercise based on HIIT would improve fitness parameters such as fat mass, blood pressure, VO_2_max, and muscle endurance following a 12-week intervention compared to changes achieved using a running-based HIIT. The purpose of this study was to investigate the effects of different kinds of training on fitness parameters in untrained female university students. It was hypothesized that (a) aerobic fitness would be increased in both the HIIT-F and HIIT-R groups; (b) that fat mass would be decreased in both the HIIT-F and HIIT-R groups; and (c) that muscular strength and endurance would be improved in the HIIT-F group.

## 2. Materials and Methods

### 2.1. Participants

Twenty untrained healthy females who were physical inactive volunteered to participate the study. Participants who did not exercise for more than 2 h weekly for at least 12 months were considered as physically inactive [31]. All of the participants were in their second year of a non-physical education-related degree at Ningbo University. Similar self-reported menstrual cycles were required, ensuring the simultaneity of testing and training. Interventions were suspended for 1 week during menstruation, and the normal menstruation period lasted for 3 to 10 days [32,33]. A randomized controlled research design was utilized, and participants were randomly assigned into a running-based HIIT (HIIT-R) (*n* = 10) or a functional training-based HIIT (HIIT-F) (*n* = 10). The participants were nonsmokers and were instructed to maintain their normal dietary intake and lifestyle habits (sleep, sit, and physical activity) throughout the intervention. Nutritional supplements and intense exercise beyond their usual exercise habits were forbidden during the intervention period [31]. All of the participants were fully familiarized with the test procedures and data collection methods prior to the intervention. Written informed consent was provided by all participants. The study was approved by the Ningbo University ethics committee. The characteristics of the participants at baseline are detailed in Table 1.

### 2.2. Procedures

A randomized controlled trial was used in this study. Each participant completed twelve weeks of 36 sessions of HIIT-R or HIIT-F intervention (three sessions per week) comprising a total of 19 min per session (10 min warm-up, 4 min work-out, and 5 min cool-down). All sessions were conducted and monitored at the same indoor stadium and at the same time of day between 9:00–10:00 a.m. Heart rates (HR) were collected with an activity wristband (Mi Smart Band 5, Xiaomi, Beijing, China) during each session to ensure that the required high intensity was achieved. The reliability and validity of the heart rate index and distance index were reported in a previous study [34]. The activity wristband was required to be worn tightly on the participant’s wrist. The HR index was measured based on changes in the light transmittance caused by blood flow density using optical sensing technology, and the distance index was measured by a triaxial acceleration sensor. Two measurement time points (pre- and post-intervention) were included. The participants were instructed to abstain from drugs, alcohol, and intense exercise two days prior to the baseline and post-intervention measurements. On the first measurement day, the participants presented themselves at 8:00 a.m. and underwent a body composition analysis, physical, and physiology measures as well as resting heart rate (HRresting) and blood pressure (BP) measurements under standardized conditions. The aerobic fitness assessment was conducted using a 12 min running test, which was completed on two days, with 24 h observed between each test. The first running test was scheduled on the first measurement day following the completion of all of the other tests, and the second trial was 24 h later. The average of the two data sets was used to assess aerobic fitness. After resting for a week [35], both groups began the training intervention. Post-intervention measurements were performed using the same methodologies as at baseline and were undertaken two days following all of the training sessions [11]. During the intervention period, additional exercises including habitual training were suspended.

### 2.3. Physical, Physiological and Body Composition Assessment

Participants were instructed to arrive at the laboratory 9:00 a.m. after a normal breakfast. Before the measurements were taken, participants were asked to empty their bladder to minimize measurement errors caused by “electrically silent” [36]. Under the guidance of two skilled operators and while wearing normal PE clothing, the participants stood on a bioelectrical impedance analysis device (BIA) (MC-180, TANITA CO., Dongguan, China) and data were presented from the device’s associated software and included height, weight, waist and hip circumference, lean muscle mass, and body fat percentage. Body mass index (BMI) was obtained by dividing weight (kg) by height (m) squared. Waist-to-hip ratio (WHR) was obtained by dividing waist (cm) by hip (cm). Blood pressure and resting HR were measured using an automatic upper arm blood pressure monitor (HEM-1000, Omron, Dalian, China). The average of the two data sets was used for analysis.

### 2.4. Aerobic Fitness Test

The most reliable and effective way to measure aerobic capacity is to record each individual subject’s VO_2_max [37]. Although maximal-effort tests are commonly used to measure VO_2_max, for untrained participants, submaximal exercises can be used as a reliable measure to estimate this value. Cooper’s 12 min running test was used to assess aerobic fitness in this study. All of the participants completed two trials of the running test separated by 24 h of rest. After a 5 min warm up, the participants were required to wear an activity wristband (Mi Smart Band 5, Xiaomi, China) and commenced running on a standard 400-metre running track. Subjects were instructed to run as many laps as possible on a standard outdoor track during the 12 min test period. All of the participants were encouraged verbally and were instructed to focus on their own pace throughout the test. The experimenter verbally provided the elapsed time at 3, 6, and 9 min. At the end of the 12 min period, the experimenter called “stop”. All of the participants ceased running and stood still, until the distance achieved, and maximal heart rate (HRmax) were recorded. The HRmax displayed on the activity wristband was recorded immediately upon the cessation of exercise, and the higher value of the two trials was used for analysis. The total distance run was determined by measures obtained from the activity band. An estimated VO_2_max was calculated using Cooper’s standardized equation [38]. The calculated VO_2_max was highly correlated with the laboratory-determined one and had acceptable reliability and validity (r = 0.897) [38]. The average of the two data sets was used to determine the VO_2_max.

### 2.5. Muscle Performance Test

Muscle performance was assessed using a field-based muscle fitness test battery. Timed sit-ups, push-ups with flexed knee (modified for females), and standing broad jump were recommended by previous studies to assess muscle performance [26,39,40,41]. All of the participants were instructed to perform the tests under supervision, and the data were recorded by the same experimenter. To assess abdominal muscular performance, the participants were asked to perform as many sit-ups as possible during a one-minute test period. The number of sit-ups that were completed correctly were recorded. A sit-up that met the following criteria was recorded: the participant lay supine on the mat with their hands crossed behind their head, elbows pointed straight forward, and knees bent at 90 degrees. The ankles were firmly held by the experimenter. During the execution of the test, the participants sat up with their heads clasped in their hands, and then their elbows touched or went over the knees, and the participant went back with their shoulders touching the mat [42]. To assess upper body strength and endurance, the flexed knee push-up option was used as a gender modification [39]. A correct flexed knee push-up met the following criteria: participants knelt on the mat with their knees bent to the mat with their arms propped on the mat slightly wider than the shoulders. When the test began, the participants were instructed to lower their body by bending their arms until their elbows were bent at a 90-degree angle and their chest was placed within 2 inches of the mat, subjects then pushed up to the starting position [43,44]. The number of correctly completed push-ups during a one-minute test period was recorded as upper body strength and endurance. Finally, the standing broad jump test was used to assess the muscle power of the lower limbs. The participants wore sneakers and stood behind the starting line with their feet placed naturally at a shoulder width apart. When testing began, the participants were instructed to bend the knees, swing the arms, and jump with both feet at the same time [45]. The jumping distance measured in centimeters was recorded, and the best of three jumps was used to determine lower limb performance. All scores were compared for statistical analysis.

### 2.6. Intervention

Exercise interventions commenced one week after the last measurement day. Both the HIIT-R and HIIT-F interventions were conducted three days per week on Mondays, Wednesdays, and Saturdays for twelve weeks. If the participants were unable to attend a scheduled exercise day, the exercise was performed on the next day and was monitored by the same researcher.

Participants in the HIIT-R group were required to complete 144 repetitions of maximal shuttle running for a total exercise time of 72 min. Each bout included a 30 s maximal shuttle run between cones placed 20 m apart with a 30 s recovery period between runs. The validity and reliability of 40 m maximal shuttle run as a measure of anaerobic performance has been reported previously [46]. The participants completed 4 bouts per session over three sessions per week. Prior to the intervention, a familiarization trial was provided to acquaint the participants with the training procedure. Running and recovery times were recorded manually using a digital stopwatch by the same experimenter. Participants were encouraged to run at their individual maximal speed for each bout.

Participants in the HIIT-F group performed multiple functional exercises using their own body weight based on Tabata training [47]. According to a recent study [48], eight movements were implemented in each session (Table 2). Participants were motivated to complete as many repetitions of a given movement as possible over 20 s followed by a 10 s recovery in the form of low intensity stepping. There was no rest period between each movement. The total training time for each session was 4 min.

The training frequency was the same as the HIIT-R group. All training exercises were recorded by video, which was provided to the HIIT-F participants prior to intervention to ensure that they were familiar with the movements and procedures. This video was played on a screen during the training intervention to ensure that the participants kept up with the rhythm of each movement.

To ensure that the interventions were performed at adequate exercise intensity, participants’ HRs were recorded throughout the session with an activity wristband. The peak heart rate (HR peak) of each session was considered to be 75% or more of the HRmax that had been recorded during Cooper’s 12 min running test. All of the sessions began with a standardized 10 min low-to-moderate running and stretching followed by maximal shuttle run or functional training and ended with a 5 min cool-down and stretching.

### 2.7. Statistical Analyses

Statistical analyses were performed using SPSS, version 23.0 (Chicago, IL, USA). Data were presented as means x± SD. A two-factor analysis of variance with repeated measures was used to analyze differences in body composition, muscle performance, and aerobic capacity, with intervention (pretraining and post training) as a within-group factor and group (HIIT-R and HIIT-F) as a between-group factor. A significant intervention x group interaction was used to identify training-induced changes in body composition, muscle performance and aerobic capacity. Data were subsequently checked by Tukey’s post hoc test if a significant interaction was revealed. Furthermore, paired t-tests were used to estimate within-group effects, and independent t-tests were conducted to examine differences between groups. The significance level was established as *p* < 0.05.

## 3. Results

All of the participants completed all of the sessions over the twelve-week period. There were no significant between-group differences in the variables measured at baseline (Table 1).

- Body Composition

**Figure 1:**
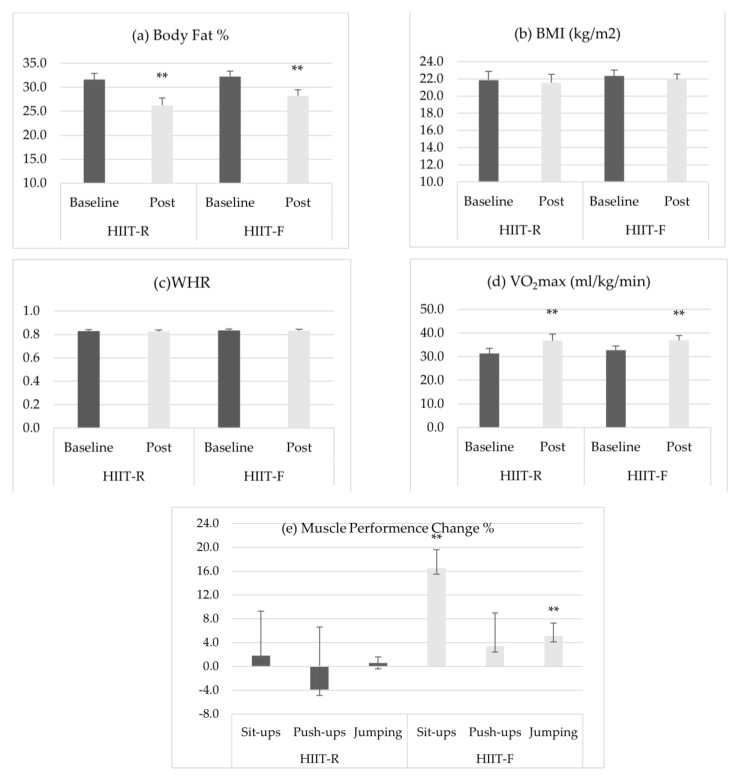
Changes in (**a**) BMI, (**b**) body fat%, (**c**) WHR, (**d**) VO2max, and (**e**) muscle performance change. Note: ** significantly different from baseline at *p* < 0.01.

Body composition data are presented in Table 3. There was a significant decrease (17.4% ± 7.4% for HIIT-R and 12.6% ± 5.1% for HIIT-F, *p* < 0.05) in the percent body fat for both groups (Figure 1a), with no interaction effect between HIIT-R and HIIT-F (*p* > 0.05). Body mass index (BMI) (Figure 1b) and waist hip ratio (WHR) (Figure 1c) did not change in either intervention (*p* > 0.05). Lean muscle mass increased in both groups (1.8% ± 1.4% for HIIT-R and 1.2% ± 1.2% for HIIT-F, *p* < 0.05).

- Resting Heart Rate and Blood Pressure

Resting HR (*p* < 0.05) was improved compared to baseline in both intervention groups, while no interaction effect was observed. Resting systolic BP and diastolic BP remained unchanged (*p* > 0.05) after training in both the HIIT-R and HIIT-F groups.

- Aerobic Capacity

VO_2_max data was calculated from the following Cooper’s equation: VO_2_max (mL/kg/min) = (distance(m)-506)/45. VO_2_max data for all participants are presented in Table 3. A significant increase (*p* < 0.05) in the VO_2_max was demonstrated in both training groups compared to baseline measures, while no significant intervention x group interaction was revealed between HIIT-R and HIIT-F after intervention compared to baseline (Figure 1c). (*p* > 0.05). The extent of the change in VO_2_max was 17.1% ± 5.6% and 12.7% ± 6.7% in the HIIT-R and HIIT-F groups, respectively.

- Muscle Performance

A significant intervention x group interaction displayed significant changes in the HIIT-R and HIIT-F groups in terms of measures of abdominal and lower limb strength (Figure 1d). In the HIIT-F group, repetitions completed during the one-minute sit-up test increased (*p* < 0.05) by 16.5% ± 3.1% and the distance obtained in the stand broad jumping test improved (*p* < 0.05) by 5.1% ± 2.2%, whereas these variables were unaltered (*p* > 0.05) in the HIIT-R group. Flexed push-ups were unaltered in both the HIIT-R and HIIT-F groups (Table 4).

## 4. Discussion

The present study aimed to investigate the effects of running and functional high-intensity training on body composition, aerobic capacity, and muscle fitness. The primary finding was that high-intensity functional training was as effective as high-intensity interval running for aerobic capacity and body composition promotion in healthy inactive females, and moreover, it induced a significant improvement in muscle fitness. The validity of this finding is supported by the fact that the mean heart rate of all of the participants reached 75% VO_2_max or above throughout the intervention. Increases in resting heart rate were also detected after training in both groups.

### 4.1. Body Composition

Our findings that HIIT-R and HIIT-F had positive effects on body composition promotion regarding the reduction of the body fat percentage were consistent with other researchers. A previous study [49] showed improved body mass, BMI, and percent body fat among obese females after a total of 108 min HIIT-R. Similarly, previous research [50] found that HIIT-R was effective in reducing BMI and body fat percentage in overweight adults. Additionally, for individuals with normal BMI, body composition improved by decreasing fat mass and increasing lean mass after a 6 -week HIIT-R intervention [51].

Not surprisingly, body composition benefits were also found in other studies investigating HIFT. Improved body fat percentage was reported after a 5-week, thrice weekly HIFT intervention [25], and further studies have also indicated a beneficial influence of HIFT on body composition [52].

However, current research has indicated that body fat percentage was significantly improved after an eight-week HIFT, while body mass was unaltered [31]. Likewise, after 16 weeks of HIFT, a significant decrease in body fat percentage was observed with no changes in the body mass [26]. Previous HIIT-R studies have provided similar results [53,54]. These results are consistent with our findings that although body fat percentage was improved, body mass and BMI were not affected by the intervention. The improved body fat percentage may be explained by the significant increase in lean muscle mass (*p* = 0.001for HIIT-R and *p* = 0.006 HIIT-F) without significant changes in the body mass (*p* = 0.064 for HIIT-R and *p* = 0.051 for HIIT-F). The non-significant change in BMI may be due to the following reasons: the insufficient exercise duration per session (2 min vs. 6–10 min); the uncontrolled dietary intake during the intervention; and the characteristics of the participants regarding body weight. This suggestion has been highlighted in a recent systematic review [55] that indicated that for normal weight populations, low-volume HIIT is inefficient for body composition improvement. Furthermore, several studies have indicated that HIIT-R and HIFT have a more significant effect on weight loss or body fat loss among obese individuals [41,48,49,50].

Finally, no significant interaction effect was revealed for any body composition variables. This suggests that HIIT-R and HIIT-F were equally effective in the modulation of body fat percentage.

### 4.2. Aerobic Fitness

VO_2_max was assessed in the present study to estimate the effects of HIIT-R and HIIT-F protocols on aerobic fitness. Running-based HIIT has been shown to increase aerobic capacity in numerous previous investigations. Several studies have reported significant increases in VO_2_max after HIIT [11,12,55]. Furthermore, a systematic review also showed that HIIT was beneficial for aerobic fitness improvements among healthy young people [56]. Nevertheless, there has been no consensus on the effect of HIFT on aerobic capacity. Some studies investigating HIFT have shown an improvement in VO_2_max [35,52,57]. On the contrary, recent research has only found aerobic capacity improvement in underweight and overweight boys, with no changes being found among normal weight people [39]. Similarly, no significant changes in VO_2_max were found after a 6-week HIFT protocol [58,59].

In our study, participants from both the HIIT-R and HIIT-F groups experienced improvements in VO_2_max (17.1% ± 5.6% and 12.7% ± 6.7%, respectively). In line with the magnitude of our results, an increase of 8% in the VO_2_max was found after a low-volume HIFT [28]. It should be noted that in the current study, the enhanced VO_2_max observed in the HIFT group was significantly higher than values recorded in previous studies. VO_2_max has been reported to improve by 5% after a HIFT with no aerobic exercise [60]. Another study showed a moderate improvement in the VO_2_max of 6.3% [31]. In our study, the greater response of VO_2_max to HIFT could be explained by the following reasons: firstly, improvements in VO_2_max were related to the testing modality [61]. Cooper’s 12 min run test demonstrated a systematic bias in favor of higher-scoring individuals [62]; secondly, this study used a longer duration (12 weeks vs. 6–8 weeks) for the implementation of functional exercises. Short or low-volume training reported no improvements in aerobic capacity, which was shown to require continuous training [14,63]. However, other investigations reported that the extent of improvement was not clearly related to training duration but to training intensity [56,64]. Therefore, further studies are required to investigate the effectiveness of the duration (work bouts/total work duration) and intensity on training-induced aerobic capacity improvement; finally, the magnitude of the improvement in VO_2_max can be attributed to the fatigue index, which was not measured in our study [11].

Although high-intensity running and functional training were both beneficial for aerobic capacity promotion, few studies have compared the effectiveness of these two exercise modalities in terms of aerobic capacity enhancement. In the current study, we controlled for the same intervention intensity and duration and found that surprisingly, there was no significant difference in terms of the changes in VO_2_max between the HIIT-R and HIIT-F groups. It is worth noting that running showed higher oxygen consumption for the same intensity compared to other modalities [65]. Our findings were partially in line with a previous study [66] that indicated no significant differences in VO_2_max promotion between high-intensity cycling and HIFT. The results from the present study illustrate that functional training is as effective as running for aerobic fitness improvement when performed at high intensity with the same volume and intensity.

### 4.3. Muscle Performance

Importantly, the repetition of sit-ups and the distance of the standing broad jump were significantly increased after HIIT-F, whereas both parameters remained unaltered in the HIIT-R group. Moreover, significant interaction effects were observed in terms of the effects on abdominal and lower limb strength and duration. Our finding is consistent with other HIFT studies. A significant increase in muscle performance after 6 weeks of HIFT was reported, whereas no increase was found in HIIT group using rowing as the exercise modality [27]. Significant improvements in lower body strength and power among patients and Army personnel were also evident [24,25]. Likewise, a study with female participants compared the effects of HIFT and endurance treadmill training on muscle fitness and demonstrated that sit-ups, chest presses, and push-ups improved by 64%, 207% and 135%, respectively, in the HIFT group after 4 weeks of intervention [24].

It is worth noting that the number of flexed push-ups that was completed in the repetition exercise was unchanged in both groups. The unchanged results are in contrast with findings from other investigations. Findings from recent studies revealed increased upper body strength and endurance after functional training executed at a high intensity [24,26,27,28]. It was possible that the observed unvaried parameters were the consequence of insufficient movements during our functional training, which lacked upper body adaptations [26]. Additionally, the assessment methods used in the present study could have also induced unaltered results. Although the flexed push-ups had been modified for females and even though the participants were familiarized with testing procedures, the participants in the present study had no or little experience and were not familiar with this movement. Furthermore, they had no knowledge of specific strategies that could be used to maximize their performance.

The effects of HIFT on muscle performance varies across exercise design and test methods. HIIT significantly increases the proportion of type I fibers [67], while muscle adaptions are specific to the exercise modality. A previous study revealed that compared to high-intensity interval running, strength training with functional movements resulted in type I muscle fibers increasing in size and a higher percentage of type IIA muscle fibers [68]. In the present study, functional exercise was more effective in strengthening muscle power than running when both were performed at relatively the same high intensity and for the same duration. However, further studies are required to investigate the training-induced individual changes in the type and size of muscle fibers between participants. Additionally, the functional exercise design should consider the fitness of the participants to reduce muscle soreness, and a previous study reported no injuries using this methodology [31].

A general limitation in the HIFT investigation was the different types of functional exercises that were included. The results might be dissimilar if HIFT was performed with other combinations of movements. Furthermore, the results of our study came from a small sample size and a non-exercising control group was not used. Finally, dietary intake was not controlled during the intervention, and the total calories consumed were not calculated. In addition, the fatigue index was not measured during the aerobic test.

## 5. Conclusions

Twelve weeks of high-intensity training based on running or functional exercises were both effective in reducing body fat percentage and improving aerobic capacity among healthy inactive females. Relative to running-based high-intensity training, HIFT shows an equally effective alternative with more exercise enjoyment and much stronger adherence regarding body composition and aerobic fitness promotion. Additionally, HIFT resulted in greater muscle performance increases than running-based high-intensity training, after which no gains were observed in terms of muscle fitness.

HIFT with self-selected intensity represents an alternative to high-intensity interval running for eliminating exercise barriers for physical exertion. Furthermore, HIFT can be performed anywhere at any time, which limits the barriers of lacking time/money. Finally, HIFT reveals strong exercise adherence and more enjoyment among females. It may be helpful for individuals to promote physical activity and the associated benefits of a prolonged healthy lifestyle.

## Figures and Tables

**Table 1:** Baseline characteristics of HIIT-R and HIIT-F group.

Parameter	HIIT-R Group (*n* = 10)	HIIT-F Group (*n* = 10)	*p*-Value
Age (yrs)	20.7 ± 0.6	20.2 ± 0.7	*p* = 0.14
Height (m)	161.1 ± 3.1	160.7 ± 2.8	*p* = 0.76
Weight (kg)	56.6 ± 6.7	57.8 ± 6.7	*p* = 0.69
Lean muscle mass (kg)	36.5 ± 1.7	36.0 ± 2.1	*p* = 0.53
BMI (kg/m^2^)	21.9 ± 3.1	22.4 ± 2.2	*p* = 0.70
WHR	0.80 ± 0.0	0.80 ± 0.0	*p* = 0.79
Body fat (%)	31.6 ± 4.1	32.3 ± 3.6	*p* = 0.71
HR resting (bpm)	70.8 ± 13.9	72.5 ± 11.2	*p* = 0.77
HR max (bpm)	188.2 ± 9.7	189.1 ± 10.4	*p* = 0.18
VO2max (mL/kg/min)	31.3 ± 7.0	32.8 ± 5.4	*p* = 0.61

Notes: BMI, body mass index; bpm, beats per minute, HIIT-F, functional exercise-based high-intensity interval training; HIIT-R, running-based high-intensity interval training; HR resting, resting heart rate; HR max, maximal heart rate; VO_2_max, maximal oxygen uptake; WHR, waist to hip ratio; yrs, years old.

**Table 2:** Details of the functional high-intensity interval training intervention.

Duration	Frequency	Exercises	Exercise Bout/Recovery Duration
12 weeks	3 sessions/week	Jumping Jacks	20 s
		Stepping	10 s
		High knees	20 s
		Stepping	10 s
		Side to side squat	20 s
		Stepping	10 s
		Mountain climbers	20 s
		Stepping	10 s
		Forearm plank to high plank	20 s
		Stepping	10 s
		Burpees	20 s
		Stepping	10 s
		Deep squat jumps	20 s
		Stepping	10 s
		Butt kickers	20 s
		Stepping	10 s

**Table 3:** Body composition and aerobic capacity data from HIIT-R and HIIT-F groups.

	HIIT-R Group (*n* = 10)				HIIT-F Group (*n* = 10)				Interaction Effect	
Parameter	Baseline	Post	Δ	*p*-Value	Baseline	Post	Δ	*p*-Value	*p*-Value	𝜂^2^
Weight (kg)	56.6 ± 6.7	55.8 ± 6.5	−1.3% ± 2.1%	ns	57.8 ± 6.7	56.6 ± 6.4	−1.9% ± 3.0%	ns	ns	0.020
Lean muscle mass (kg)	36.5 ± 1.7	37.2 ± 1.8	1.8% ± 1.4%	*p* < 0.05	36.0 ± 2.1	36.4 ± 2.1	1.2% ± 1.2%	*p* < 0.05	ns	0.056
BMI (kg/m^2^)	21.9 ± 3.1	21.6 ± 3.1	−1.3% ± 2.1%	ns	22.4 ± 2.2	21.9 ± 2.1	−1.9% ± 3.0%	ns	ns	0.018
WHR	0.8 ± 0.0	0.8 ± 0.0	−0.6% ± 0.9%	ns	0.8 ± 0.0	0.8 ± 0.0	−0.3% ± 0.5%	ns	ns	0.032
Body fat (%)	31.6 ± 4.1	26.3 ± 4.8	−17.1% ± 7.4%	*p* < 0.01	32.3 ± 3.6	28.3 ± 3.9	−12.6% ± 5.1%	*p* < 0.01	ns	0.118
HR resting (bpm)	76.5 ± 10.1	74.2 ± 7.4	−2.5% ± 5.5%	ns	77.8 ± 9.1	75.3 ± 8.6	−3.1% ± 5.3%	ns	ns	0.001
HR max (bpm)	188.7 ± 6.7	185.8 ± 6.0	−1.5% ± 1.1%	*p* < 0.05	183.7 ± 9.3	181.8 ± 7.8	−1.0% ± 1.3%	*p* < 0.05	ns	0.050
VO2max (mL/kg/min)	31.3 ± 7.0	36.7 ± 8.8	17.1 ± 5.6%	*p* < 0.01	32.8 ± 5.4	36.9 ± 6.4	12.7% ± 6.7%	*p* < 0.01	ns	0.075

Note: Δ (post-baseline)/baseline; ns, no significance; partial 𝜂^2^ value for effect size.

**Table 4:** Muscle performance data from HIIT-R and HIIT-F groups.

	HIIT-R Group (*n* = 10)				HIIT-F Group (*n* = 10)				Interaction Effect	
Parameter	Baseline	Post-Training	Δ	*p*-Value	Baseline	Post-Training	Δ	*p*-Value	*p*-Value	𝜂^2^
Sit-ups (reps)	35.3 ± 6.7	35.7 ± 5.9	1.8% ± 7.5%	ns	37.3 ± 4.8	43.4 ± 5.3	16.5% ± 3.1%	*p* < 0.01	*p* < 0.01	0.760
Flexed push-ups (reps)	7.7 ± 1.3	7.4 ± 1.6	−3.9% ± 10.5%	ns	8.0 ± 1.4	8.3 ± 1.7	3.4% ± 5.6%	ns	ns	0.180
Standing broad jump (cm)	176.0 ± 5.8	177.1 ± 5.5	0.6% ± 1.0%	ns	178.0 ± 6.1	187.0 ± 5.5	5.1% ± 2.2%	*p* < 0.01	*p* < 0.01	0.686

Note: Δ (post-baseline)/baseline; ns, no significance; partial 𝜂^2^ value for effect size.

## Data Availability

The data presented in this study are available upon request from the corresponding author. The data are not publicly available due to student privacy.
